# Perceived Attitudes About Substance Use in Anonymous Social Media Posts Near College Campuses: Observational Study

**DOI:** 10.2196/mental.9903

**Published:** 2018-08-02

**Authors:** Alexis S Hammond, Michael J Paul, Joseph Hobelmann, Animesh R Koratana, Mark Dredze, Margaret S Chisolm

**Affiliations:** ^1^ Department of Psychiatry and Behavioral Sciences The Johns Hopkins University Baltimore, MD United States; ^2^ Department of Information Science University of Colorado, Boulder Boulder, CO United States; ^3^ Department of Computer Science Stanford University Stanford, CA United States; ^4^ Department of Computer Science The Johns Hopkins University Baltimore, MD United States

**Keywords:** Yik Yak, college, social media, substance, drugs, alcohol

## Abstract

**Background:**

Substance use is a major issue for adolescents and young adults, particularly college students. With the importance of peer influence and the ubiquitous use of social media among these age groups, it is important to assess what is discussed on various social media sites regarding substance use. One particular mobile app (Yik Yak) allowed users to post any message anonymously to nearby persons, often in areas with close proximity to major colleges and universities.

**Objective:**

This study describes the content, including attitude toward substances, of social media discussions that occurred near college campuses and involved substances.

**Methods:**

A total of 493 posts about drugs and alcohol on Yik Yak were reviewed and coded for their content, as well as the poster’s attitude toward the substance(s) mentioned.

**Results:**

Alcohol (226/493, 45.8%), marijuana (206/493, 41.8%), and tobacco (67/493, 13%) were the most frequently mentioned substances. Posts about use (442/493) were generally positive toward the substance mentioned (262/442, 59.3%), unless the post was about abstinence from the substance. Additionally, posts that commented on the substance use of others tended to be less positive (18/92, 19.6% positive) compared to posts about one’s own use (132/202, 65.3% positive).

**Conclusions:**

This study provides a description of anonymous discussions on or near college campuses about drugs and alcohol, which serves as an example of data that can be examined from social media sites for further research and prevention campaigns.

## Introduction

Substance use is a major public health problem for adolescents and young adults in the United States, and college students have their own particular set of risks for use and barriers to treatment [1,2]. Of an estimated 9 million full-time college students in 2014, approximately 10% reported initial use in the past year of alcohol and 6% reported initial use of illicit drugs (including marijuana, which accounted for the vast majority of this category [3]). Approximately 20% reported using illicit drugs in the past month, almost 40% reported binge drinking, and 13% reported smoking cigarettes. Substance use (particularly alcohol use) has become normative in the college culture, and the influence of peers is certainly a major factor affecting this stage of development [1].

The use of social media among adults aged 18 to 29 years is now essentially ubiquitous; 90% reported use in 2015 compared to 12% in 2005 [4]. This use has expanded the social network of young adults, and online platforms may have more influence on their substance use than actual in-person interactions [5,6]. Historically, college students have perceived that their peers used substances more than they actually do [7-9]. As such, exposure to substance use via social media may normalize the use for some young adults by presenting it in a positive light, potentially providing a skewed perspective compared to the actual behavior of most college-aged persons. In fact, a study involving the social networking site (SNS) Twitter showed that messages (“tweets”) about alcohol use (especially heavy use and binge drinking) are quite common and typically portray a positive attitude about the substance (pro-alcohol tweets were 10 times more frequent than anti-alcohol tweets [10]). Another study examined posts about marijuana on the SNS Instagram, which tends to have younger users (approximately 40% of users are younger than age 24 years), and found that the majority of these posts were also pro-use [11]. Finally, a study examining tweets about menthol cigarettes reported more complex findings, with 48% of tweets being positive and 40% negative, but negative views were common among nonsmokers (91%) and former smokers (71%) [12]. Topics associated with negative sentiment included health and smoking cessation. Together, these findings demonstrate the large variety of substance-related topics discussed on social media sites, which can be a source of data for substance use research, and the complexities of the discussions, which are dependent on the substance mentioned and the perspective of the poster.

Previous studies have shown that substance use rates tend to be higher when collected via self-report methods with some anonymity built in versus direct interviews [13-16], indicating a preference for privacy when discussing this sensitive topic. Accordingly, although some users seem to be open about what they post online, it is possible that certain topics related to substance use may not be as readily discussed by all persons on public sites due to concerns about issues such as privacy or stigma. Thus, it would be interesting to understand whether an added layer of privacy changes the content of posts. The social media mobile app Yik Yak, which was in use from November 2013 until the company ceased operations in May 2017, initially differed from sites such as Facebook because it allowed users to post messages (“yaks”) anonymously, without any form of personal identifiers such as usernames, which limited traceability. This was somewhat similar to websites such as Drugs-Forum.com and BlueLight.com, where individuals have partial anonymity (by use of pseudonym usernames) and share information about how to prepare and administer certain drugs, as well as potential effects (positive or negative) to expect. Like Twitter, there was a character limit; Yik Yak had a 200-character limit for each yak. Another difference between Yik Yak and other sites was Yik Yak’s restriction of user interaction to a 5-mile radius. Thus, users were only able to communicate with persons that were nearby. The locations popularly served by Yik Yak tended to center around college campuses and, as anyone within a specific area could participate in the network by responding to the post, this effectively targeted a particular audience. Being able to post anonymously to nearby persons not only allowed for free discussion of sensitive topics, such as substance use, but also helped facilitate in-person interactions of people with similar interests. Although these features may have led to the controversial situations and negative press involving Yik Yak due to reports of cyberbullying with subsequent discontinuation of the app [17], research involving SNS or apps with these characteristics provides an opportunity for observation of attitudes and potential practices related to substance use among college students. SNS are very diverse and constantly changing, and it is important to capture information from different sites at varying times to ensure reliability and ease of replication to inform future research.

We previously reported on the types of topics discussed on Yik Yak near college campuses, with a particular focus on general health-related topics [18]. Yik Yak was chosen for study due to the lack of research on it and its features of anonymity and geospatial restriction. We noted that a large share of posts were about sensitive topics such as sex and drugs, which may be related to the anonymity of the platform. Further analysis of substance-related posts showed that most were related to buying substances. In this study, our objective was to further characterize posts that mentioned licit and illicit substances, categorizing them by types of posts, substance(s) mentioned, and poster attitudes toward specific substances while identifying the frequencies of these characteristics. We hypothesized that posts about one’s own use of substances would be more likely to result in a categorization implying a positive attitude compared to posts about others’ use of substances.

## Methods

The dataset was created by downloading messages from Yik Yak. We created a tool that emulated the protocol that mobile devices would use to communicate with Yik Yak servers. This tool allowed us to programmatically retrieve and store yaks in real time into a database for further processing on our end. Additionally, we were able to use the developed tool to change the target location to collect yaks from a variety of locations. We continuously polled the incoming posts, comments, and their respective latitude and longitude for our dataset. The tool allowed us to download messages within a 5-mile radius of a provided latitude and longitude. We used this tool to collect yaks near 120 college campuses in the United States. This set of campuses included the largest universities in the United States, along with additional universities that we included to increase the breadth and diversity of our collection—based on academic rigor, culture, and politics of the location—and also included universities in population-dense cities. For each campus, we queried for yaks within the radius of the campus’ geocoordinates, which we obtained from the Google Maps Geocoding API [19]. We downloaded yaks from June 12, 2015 to July 14, 2015, an arbitrary period based on the initial development of our collection tool. We stopped scraping the yaks as our sample size grew to a sizeable amount. The crawler software returned the 100 most recent yaks for a particular location and provided us with 122,179 total yaks.

**Table 1:** Examples of posts (“yaks”) and how they were categorized.

Code	Substance	Paraphrased Yak
Own use, positive	Alcohol	I just want to watch the game, drink some beers, and relax.
Other’s use, negative	Tobacco	Cigarette smokers: how selfish to make others breathe in your smoke!
Meeting to use, positive	Marijuana	Who is downtown and wants to smoke a joint with me?
Request for Information, neutral	Other	What’s a good price for a gram of cocaine?

Although users could also reply to yaks, we focused on the original yaks in this paper because responses were often quite varied and not always specific to the original post. This study analyzed only the data available from Yik Yak, which maintains the anonymity of the user. The Johns Hopkins University institutional review board approved analysis of publicly available social media messages.

To find yaks relevant to substances, we keyword-filtered original posts matching a large set of substance-related keywords (see Multimedia Appendix 1 for the list of keywords). We manually examined the 12,292 retrieved posts as a first pass and manually removed blatantly irrelevant messages, resulting in a dataset of 2047 substance-related yaks. To characterize the data in greater depth, we reviewed and coded a convenience sample of the first 500 yaks (from a wide variety of universities) of the sample of yaks identified as mentioning substances. Two reviewers (ASH and JGH) read each yak and confirmed whether they were indeed about substances; if they were, the substance(s) was identified and categorized as being alcohol, tobacco, marijuana, and/or other. (The majority of substances fell in the first three categories and due to the small number of other substances mentioned, such as cocaine, Adderall, and LSD, they were grouped together in the “other” category.)

Yaks were then coded for the content based on whether the post was about actual use of a substance or nonuse (typically rhetorical comments about drugs or jokes). Posts specifically about use were then categorized into one of eight types: (1) first-person accounts of use, including effects, (2) comments on use by another person, (3) obtaining substances (through buying or bartering), (4) meeting to use, (5) selling or trading, (6) abstinence from a substance (cessation or cutting down use), (7) laws about use, and (8) questions to obtain information, such as how to use certain substances. Reviewers also coded the displayed attitude (positive, negative, or neutral) of the poster toward the substance(s) mentioned. Reviewers kept this standard by focusing on whether the poster was in support of the substance mentioned specifically, and not the overall emotional affect of the post. If this was ambiguous, or the person was neither in support of nor against the substance, it was rated as neutral. (See Table 1 for paraphrased examples of yaks with codes.) When there was any discordance between the two reviewers at any step in the review and coding process, a third reviewer (MSC) resolved the discordance. The three reviewers established a codebook and discussed what content would be included among each category a priori. Data were analyzed to look at frequencies of particular posts by category and/or substance, and coding agreement among the first two raters was assessed using Cohen kappa coefficient (κ).

## Results

### Overview

Of the subset of 500 yaks, 493 yaks (98.6%) were confirmed as related to substances on the manual second pass. Although some of the 493 yaks mentioned more than one substance, alcohol (n=226), marijuana (n=206), and tobacco (n=67) were the most frequently mentioned substances. The remaining substances mentioned (n=47) were grouped together as “other” due to low frequency and included “acid,” Adderall, methamphetamines, and cocaine. The Cohen kappa score for substance was .98. In all, 53 yaks mentioned two or more substances; those most often mentioned together were alcohol/marijuana (20/53, 38%), alcohol/tobacco (12/53, 23%), alcohol/other substance (11/53, 21%), marijuana/other substance (10/53, 19%), and marijuana/tobacco (7/53, 13%).

### Content of Posts

The majority of yaks (442/493, 89.7%; Table 2) were about use of a substance (κ=.56) and, among these, 202 (45.7%) were about the poster’s own use, 92 (20.8%) were commenting on someone else’s use, 45 (10.2%) involved discussion of meeting up with someone to use, 40 (9.0%) involved buying substances, and 31 (7.0%) asked for information about using. Less common categories of use included the discussion of selling substances (12/442, 2.7%), abstinence from use (9/442, 2.0%), and the legal statuses of substances (10/443, 2.3%). The Cohen kappa score for all categories was .92.

### Attitudes of Posts

Overall, posts about substance use were mainly positive toward the substance (262/442, 59.3%; Table 3), with 79 (17.9%) being negative and 101(22.9%) neutral. Tobacco posts tended to be more negative toward the substance, while posts in the “other” category were generally spread out evenly among attitudes. Among the various “use” categories, positive posts included those about meeting up to use (45/45, 100% positive) and about one’s own use (132/202, 65.3% positive). Posts about others’ use (18/92, 20% positive) and abstinence (1/9, 11% positive) were less positive (Table 4). Posts about the legal status of tobacco were 100% negative (3/3) toward tobacco. These expected attitude trends often persisted when looking at categories of use by substance as well (data not shown), with the notable exception that only 8/32 (25%) comments about other’s use of marijuana were negative, with most being positive (10/32, 31%) or neutral (14/32, 43%). The Cohen kappa score for attitudes was .73.

**Table 2:** Categories of codes, as a function of substance mentioned.

Code		Alcohol (N=226), n (%)	Tobacco (N=62), n (%)	Marijuana (N=206), n (%)	Other (N=47), n (%)
**Use**		**195 (86.3)**	**62 (92.5)**	**193 (93.7)**	**38 (80.9)**
	First-person account of use	104 (53.3)^a^	17 (27.4)^b^	84 (43.5)^c^	21 (55.3)^d^
	Comments on others’ use	35 (17.9)^a^	31 (50.0)^b^	32 (16.6)^c^	7 (18.4)^d^
	Obtaining substance	13 (6.7)^a^	0 (0.0)^b^	22 (11.4)^c^	5 (13.2)^d^
	Meeting to use	21 (10.8)^a^	2 (3.2)^b^	24 (12.4)^c^	1 (2.6)^d^
	Selling or trading	6 (3.1)^a^	0 (0.0)^b^	6 (3.1)^c^	1 (2.6)^d^
	Abstinence	1 (0.1)^a^	7 (11.3)^b^	1 (0.05)^c^	0 (0.0)^d^
	Laws about use	1 (0.1)^a^	3 (4.8)^b^	7 (3.6)^c^	0 (0.0)^d^
	Requests for information	13 (6.7)^a^	2 (3.2)^b^	17 (8.8)^c^	3 (7.9)^d^

^a^N=195.

^b^N=62.

^c^N=193.

^d^N=38.

**Table 3:** Attitudes of posters about particular substances.

Attitude toward substance	Alcohol (n=195), n (%)	Tobacco (n=62), n (%)	Marijuana (n=193), n (%)	Other (n=38), n (%)
Positive	116 (59.5)	13 (21.0)	137 (71.0)	18 (47.4)
Negative	30 (15.4)	36 (58.1)	15 (7.8)	9 (23.7)
Neutral	49 (25.1)	13 (21.0)	41 (21.2)	11 (28.9)

**Table 4:** Attitudes of posters toward substance as a function of the selected category of use.

Attitude	Own use (n=202), n (%)	Others’ use (n=92), n (%)	Looking to buy (n=40), n (%)	Requests for information (n=31), n (%)	Abstinence (n=9), n (%)
Positive	132 (65.3)	18 (19.6)	34 (85.0)	16 (51.6)	1 (11.1)
Negative	26 (12.9)	44 (47.8)	0 (0.0)	0 (0.0)	6 (66.7)
Neutral	44 (21.8)	30 (32.6)	6 (15.0)	15 (48.4)	2 (22.2)

## Discussion

Our finding that most posts related to substances were positive is consistent with previous studies [10,20-23]. A selection bias exists in this data because these attitudes may not be consistent with the majority of college students, but the findings certainly highlight the type of content to which any college student may be exposed to via social media. A previous study identified prescription drug “abusers” on Twitter and found that persons in their social circles also tended to discuss prescription drug use online [24], providing further evidence of the influence from, and reinforcement of, online content. In fact, viewing and posting about substance use appears to correlate with actual use. Research among Twitter users found that exposure to positive messages about alcohol and marijuana was significantly associated with current heavy episodic drinking and current marijuana use, respectively [25]. Similarly, college students younger than age 21 who posted items on their Facebook profile that were related to intoxication showed higher scores related to problem drinking on the Alcohol Use Disorders Identification Test (AUDIT) scale [26] and were more likely to report having an alcohol-related injury in the past year as opposed to students who did not display references to alcohol [27]. This study was unable to correlate substance use with postings due to the anonymous nature of Yik Yak, and more research is needed to better understand and replicate this phenomenon. As new substances or substance use patterns emerge, social media sites continue to provide an opportunity for health surveillance.

Interestingly, attitudes of posts requesting information about substances, such as how to use them, were almost evenly split between positive and neutral suggesting ambivalence among some posters, which could then be influenced by responses to their posts. We did not analyze replies to original posts, although this would be an interesting avenue for future research to see how often comments agreed or disagreed with the original post or provided helpful or harmful information in response. This could also be an opportunity for intervention in the future: to dissuade young adults from initiating use of a substance or to provide evidence-based health information for this vulnerable population.

Another limitation of the study is, due to the anonymous nature of Yik Yak, we do not know any demographic characteristics of the posters, including age or student status, and have no way to tell if any postings were from automated accounts or “bots” [28]. Posts were shared within a 5-mile radius of a university at the time of posting, so this is a study of areas on and around colleges and universities, but it is not necessarily a study of college students. This information is still important due to the potential for other persons to attempt to sell substances to students, expose them to information about substance use, or meet up with them to use. Additionally, we were limited to a 1-month period of time to collect data, which fell during the summer, a time when some college students are not in town. Future studies may collect more varied information by collecting over a longer duration of time or at repeated points in time.

This study provides a glimpse into the discussion of multiple substances on or near college campuses through an anonymous social media mobile app. Social media sites are constantly changing and evolving, and it is important to collect data across different sites over varying times and durations of time to both capture information and produce reliable results that can be replicated [29]. Yik Yak had unique features, mainly being a location-oriented site with strong anonymity, and it is very likely that similar sites may be developed or those with other novel features. Thus, although Yik Yak is no longer in use and this specific study cannot be replicated, it is still important to report the methodology and findings to inform future studies among current social media sites and novel sites that are certain to arise. The data reported here provide an example of the kind of information that can be examined from publicly available social media posts that may inform health prevention and treatment strategies. For example, this kind of information may prove useful for developing public health campaigns relevant to this population, such as dispelling common myths or advising of the consequences associated with use, possession/distribution, or meeting up with strangers to use.

## Appendix

Multimedia Appendix 1 List of keywords used in Yik Yak search.

## Abbreviations

AUDIT: Alcohol Use Disorders Identification Test
SNS: social networking site
