# Supplementary material for: Perceived Attitudes About Substance Use in Anonymous Social Media Posts Near College Campuses: Observational Study
Source: JMIR Ment Health. 2018 Aug 2;5(3):e52. doi: 10.2196/mental.9903 (PMC6096169; doi:10.2196/mental.9903)
Supplement: Multimedia Appendix 1 [file mental_v5i3e52_app1.pdf]

alcohol
drink
drank
drinking
drinks
drunk
absinthe
vodka
beers
beer
alcoholic
booze
everclear
liquor
dose
drug
drugs
heroin
smoking
mdma
smoke
dxm
lsd
doses
pills
opiates
weed
meth
cocaine
kratom
cannabis
adderall
opiate
addiction
pill
acid
smoked
codeine
morphine
salvia
methadone
dosage
prescribed
tramadol
benzos
amphetamine

marijuana
mephedrone
oxycodone
substances
withdrawal
snorting
serotonin
xanax
dmt
grams
gram
pipe
opium
benzo
hydrocodone
ghb
ketamine
thc
dopamine
orally
pot
prescription
comedown
tobacco
oxy
amphetamines
psychedelic
addictive
ritalin
meds
tripping
trippin
ecstasy
bong
snort
addicted
stimulant
overdose
stimulants
fentanyl
dosing
snorted
suboxone
alprazolam
potency
methamphetamine

opioid
poppy
oxycontin
diazepam
sober
gbl
hash
withdrawals
methylone
clonazepam
hallucinations
valium
addict
cannabinoids
medications
addicts
opioids
psychedelics
ssris
dosages
stoned
ssri
alkaloids
mescaline
adhd
cough syrup
nicotine
capsules
methylphenidate
lighter
lsa
cigarettes
blotter
binge
benzodiazepines
cigarette
buprenorphine
jwh
rolling
rollin
mdpv
shrooms
erowid
joint
joints
resin

hydromorphone
ambien
5-htp
vyvanse
cannabinoid
lorazepam
2c-e
gaba
psychoactive
piracetam
junkie
klonopin
vicodin
concerta
maoi
2c-i
hangover
hungover
dexedrine
mda
antidepressants
melatonin
smoker
smokers
naloxone
subutex
potentiate
benzodiazepine
diphenhydramine
depressants
antidepressant
ayahuasca
prozac
temazepam
bupe
percocet
am-2201
dilaudid
side effects
side effect
zolpidem
dosed
insufflated
phenibut
xtc
trippy

robo
robotrip
robotripping
robotrippin
reuptake
oxys
withdrawl
blotters
detox
narcotics
kava
sublingual
redose
sativa
narcotic
divinorum
salvinorin
poppies
hydrochloride
junkies
dextromethorphan
inhibitor
downers
norepinephrine
ephedrine
meph
ativan
2c-b
vaporizer
oxymorphone
dextroamphetamine
tincture
pcp
bongs
redosing
analgesic
zoloft
peyote
zopiclone
laced
wellbutrin
dex
pharmacist
ether
skunk
blunt

dihydrocodeine
effexor
paxil
painkillers
incense
focalin
indica
depressant
serotonergic
benadryl
citalopram
bupropion
promethazine
dissociative
ecstacy
uppers
hallucinogens
hbwr
modafinil
binges
soma
hallucinogenic
ket
mirtazapine
seroquel
adderal
gabapentin
phenazepam
loperamide
maois
afterglow
blunts
hemp
san pedro
nmda
cyp2d6
spliff
venlafaxine
5-ht
percs
toke
toking
nitrous
nootropics
cigs
cbd

hangovers
robitussin
tussin
psilocybin
zicam
fluoxetine
delsym
k-hole
contin
lexapro
desoxyn
5htp
snuff
hppd
speedball
hashish
codiene
methyl
hallucinogen
hallucination
vaporizing
valerian
tryptamines
etizolam
phenethylamines
midazolam
nootropic
dopaminergic
cig
vape
mimosa
menthol
diamorphine
antihistamine
gelcaps
antihistamines
overdosing
pseudoephedrine
cb1
caapi
naltrexone
overdosed
woodrose
yopo
snri
sertraline

norco
phenethylamine
paroxetine
painkiller
dissociatives
bzp
somniferum
bufotenine
4-mmc
4-fa
oding
peruvian torch
2c-t-7
mdai
headshop
2ci
barbiturates
hallucinate
trazodone
tweaking
binging
sobriety
thebaine
opiod
papaver
k2
lighters
hallucinating
tyrosine
junky
pregabalin
opiods
percocets
munchies
drug test
drug tested
snus
gravel
alpha-PVP
flakka
